# Rejuvenating the blood and bone marrow to slow aging-associated cognitive decline and Alzheimer’s disease

**DOI:** 10.1038/s42003-020-0797-4

**Published:** 2020-02-13

**Authors:** Seokjo Kang, V. Alexandra Moser, Clive N. Svendsen, Helen S. Goodridge

**Affiliations:** Board of Governors Regenerative Medicine Institute and Department of Biomedical Sciences, Cedars-Sinai Medical Center, 8700 Beverly Boulevard, Los Angeles, CA 90048 USA

**Keywords:** Bone marrow transplantation, Haematopoietic stem cells, Cognitive ageing

## Abstract

Parabiosis, blood exchange and plasma transfer experiments have highlighted the rejuvenating properties of young blood. Our *Communications Biology* study demonstrated that young bone marrow transplantation attenuates cognitive decline in old mice, with preservation of hippocampal synapses and reduced microglial reactivity. We now discuss subsequent studies that shed additional light on how blood impacts cognitive function, and potential clinical applications, including ongoing clinical trials with young plasma and experimental strategies targeting the hematopoietic system to slow or reverse cognitive decline.

## Abstract (web summary)

Research from Helen Goodridge and Clive Svendsen’s groups published in Communications Biology showed that young bone marrow transplantation slows down cognitive decline in old mice. The authors now discuss subsequent studies examining the relationship between blood components and aging-associated cognitive impairment and rejuvenation as well as their own ongoing work.

## Preservation and rejuvenation of cognition during aging by young blood

Cumulative evidence from parabiosis, blood exchange and plasma transfer experiments has demonstrated that young blood can rejuvenate multiple organs in old mice, including the brain, liver, muscle, and heart, and that old blood accelerates aging in young mice (reviewed in refs. ^1,2^). Heterochronic parabiosis studies, in which old and young mice are surgically joined so they share a circulatory system, have suggested that circulating components of young blood can induce brain rejuvenation in old parabionts, including improvement of hippocampal learning and memory (reviewed in ref. ^3^). A single blood exchange between old and young mice (without organ sharing, which might confound parabiosis experiments) had similar outcomes^4^, and plasma transfer experiments implicated soluble factors in the circulation^5,6^.

Together these studies suggest that young and old blood contain varying levels of youthful and pro-aging factors, and that manipulation of those factors represents a promising therapeutic approach for the treatment of human aging-associated diseases such as Alzheimer’s disease^7^. However, more mechanistic insight is needed to further inform the design of effective therapeutic strategies. This includes additional information about the sources of circulating factors, which are often produced by multiple cell types (hematopoietic and non-hematopoietic), and their mechanisms of action, both systemically and in the brain.

In our *Communications Biology* study^8^, we performed heterochronic bone marrow transplantation (BMT) to determine the impact of systemic hematopoietic aging on cognitive function, neurons and glial cells. We performed behavioral testing and histological and molecular analyses 6 months after transplantation of total bone marrow cells from either young (4-month) or aged (18-month) donor mice into aged recipient mice (18 months old at the time of BMT). Young BMT preserved hippocampal learning and memory in the old mice (24 months old at the time of testing), prevented loss of synaptic connections and reduced microglial activation in the hippocampus. In addition, our data implicated increased circulating levels of the chemokine CCL11 in microglial activation and synaptic disruption in the hippocampus of old mice, and consistent with the preservation of cognitive function, we observed reduced levels of circulating CCL11 in young bone marrow recipients. We thus demonstrated that aging-associated cognitive decline is at least partially due to aging of the peripheral hematopoietic system and perhaps specifically linked to CCL11.

Since the publication of our paper, several other studies have described the relationship between blood components and aging-associated cognitive impairment and rejuvenation. These are reviewed below in relation to our ongoing studies aimed at better understanding this fascinating phenomenon.

## Recent insights into how blood influences brain aging and rejuvenation

A recent paper by Gan and Sudhof demonstrated that factors in young mouse blood can directly promote synaptic function^9^. They first examined the effect of serum from young and old mice on cortical neurons derived from human embryonic stem cells and found that young, but not old, serum promoted synapse formation, increased neurotransmitter release, and enhanced recruitment of synaptic N-methyl-D-aspartate receptors, resulting in elevated synaptic connectivity. They identified two factors—THBS4 and SPARCL1—enriched in serum from young mice that act directly on neurons as synaptogenic factors. Treatment of cultured neurons with recombinant THBS4 and SPARCL1 boosted synapse formation in vitro. However, as the researchers noted, further study is required to clarify whether these factors are capable of crossing the blood-brain barrier, and whether they could recover neuronal synaptic activity in vivo.

Another recent study implicated vascular inflammation in the effects of old blood on the aging brain. Yousef et al. reported that acceleration of brain aging by old plasma depends on increased expression of vascular adhesion molecule 1 (VCAM1) on the luminal side of brain endothelial cells (BECs) that line the blood-brain barrier^10^. Systemic administration of anti-VCAM1 antibody and deletion of the *Vcam1* gene in BECs reversed features of brain aging, including microglial reactivity and lower neural precursor cell activity, and rescued cognitive function in aged mice. The authors thus proposed a model in which inflammatory cytokines in old plasma increase VCAM1 expression on BECs, thereby promoting leukocyte tethering but not transmigration, resulting in BEC inflammation, which in turn activates microglia and hinders neurogenesis.

Aging is the greatest risk factor for a number of neurodegenerative diseases including Alzheimer’s disease. Xia et al. recently demonstrated that intravenous delivery of serum from young mice ameliorates cognitive decline in the APP/PS1 mouse model of Alzheimer’s disease, in part by restoring the hippocampal cholinergic circuit^11^. Impaired hippocampal cholinergic innervations and reduced levels of phosphorylated-M1 muscarinic acetylcholine receptor (M1 mAChR) in APP/PS1 mice were rescued by intravenous injection of young serum (100 μl per injection, 10 times over 30 days), but these neuroprotective effects were abrogated by benztropine, an M1 mAChR inhibitor. The rejuvenating effects of young serum in APP/PS1 mice were partially achieved by activation of REST/FOXO1 signaling, which mediates stress resistance against toxic insults and maintains neuronal health.

## Plasma transfer in clinical trials

Findings from studies like ours are already being translated in clinical settings. One example of this is a current clinical trial based on the pre-clinical work of Wyss-Coray and colleagues, who found that administration of young plasma into old mice led to increased synaptic plasticity and improvements in cognitive performance^5,6^. These findings were replicated in a mouse model of Alzheimer’s disease, where it was shown that young plasma restored expression of synaptic and neuronal markers, and improved performance in two memory tasks^12^. These effects were achieved without any alterations in amyloid-β plaque deposition, the pathological hallmark of Alzheimer’s disease, suggesting that young plasma acts on non-amyloidogenic pathways.

On the basis of these pre-clinical findings, Alkahest has initiated several clinical trials for Alzheimer’s and Parkinson’s diseases (NCT03520998, NCT03713957, NCT03765762), macular degeneration (NCT03558061, NCT03558074), and postoperative recovery following knee or hip replacements (NCT03981419). Earlier this year, Alkahest reported findings from their first clinical trial (NCT02256306) in patients with mild to moderate Alzheimer’s disease^13^. The 4 weekly infusions of young plasma (~250 ml each) were well-tolerated and safe, but the small sample size did not allow for determination of efficacy. Phase 2 clinical trials for both mild to moderate and severe Alzheimer’s disease are ongoing.

Additional ongoing clinical trials are administering young plasma to Parkinson’s disease patients in the United States (NCT02968433), umbilical cord blood and plasma for treatment of aging-associated frailty in South Korea (NCT02418013), and young plasma to improve neurologic outcomes after acute stroke in a Chinese trial (NCT02913183).

Although the potential of young blood products to improve aging-associated decline and diseases is being legitimately studied in several clinical trials, some companies have sought to rapidly monetize this field without having performed the necessary safety and efficacy studies. One of these companies, Ambrosia, began running a “pay-to-participate” clinical trial using young plasma in 2016. Though the company’s founder has touted numerous benefits ranging from improved cognition to reduced blood cholesterol (https://www.newscientist.com/article/2133311-human-tests-suggest-young-blood-cuts-cancer-and-alzheimers-risk/), data from this study remains unpublished. Ambrosia shut down in February 2019, shortly after the FDA released a statement warning of the lack of proven benefits as well as potential dangers associated with young plasma infusions (https://www.fda.gov/vaccines-blood-biologics/safety-availability-biologics/important-information-about-young-donor-plasma-infusions-profit).

There is great excitement and certainly some potential for these sorts of therapies to alleviate aging-associated disorders, but rigorous scientific and clinical trials are needed to evaluate their safety and verify their effects.

## Alternative translational opportunities

**Fig. 1:**
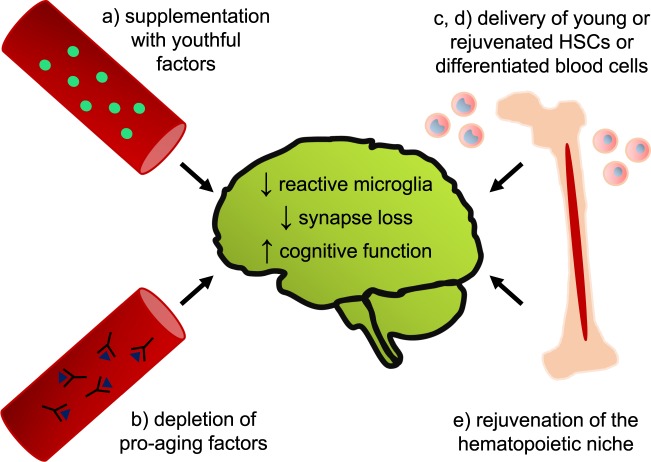
Potential peripheral interventions to prevent or rescue aging-associated cognitive decline. A greater understanding of the impacts of circulating factors on the hippocampus may inform strategies to replace youthful factors lost during aging (**a**) or neutralize pro-aging factors (**b**). Alternatively, it may be more effective to deliver young or rejuvenated HSCs (**c**) or differentiated hematopoietic cells (**d**), or to target the bone marrow niche to rejuvenate endogenous HSCs (**e**).

As an alternative to plasma transfer, we are exploring five potential rejuvenation strategies to slow aging and ease the burden of neurodegenerative disease in humans: delivery of youthful factors, neutralization of pro-aging factors, young hematopoietic stem cell (HSC) transplantation, delivery of specific subsets of differentiated hematopoietic cells, and rejuvenation of endogenous HSCs (Fig. 1).

## Delivery of youthful factors to promote cognitive health

Plasma transfer experiments demonstrating rejuvenation following intravenous delivery of a relatively small volume of young plasma or serum into old mice (100–175 μl, 6–10 times over 2–4 weeks)^5,6,11,12^ suggest that supplementation with youthful factors may be sufficient to promote cognitive health, both during healthy aging and in neurodegenerative disease. Systemic administration of Growth Differentiation Factor 11 (GDF11) increased the number of neural stem cells and improved vascular remodeling in the subventricular zone and hippocampus of aged mice^14,15^. GDF11 may not cross the blood-brain barrier but instead impact the brain parenchyma by enhancing vascular endothelial growth factor (VEGF) secretion by brain vascular endothelial cells^15^, thereby stimulating both angiogenesis and neurogenesis^16^. THBS4 and SPARCL1 may also be beneficial^9^ if these factors are capable of crossing the blood-brain barrier to promote synapse formation and function.

## Neutralization of pro-aging factors to prevent or reverse cognitive decline

Alternatively, it may be necessary to neutralize the effects of pro-aging factors like CCL11, TNF-α and IL-1β, perhaps using antibodies, antisense oligonucleotides, or receptor antagonists. More research is needed to identify candidates and evaluate how these factors could be targeted. It would likely be easier to target factors produced in the periphery than factors produced in the brain. It will also be important to understand whether circulating pro-aging factors cross the blood–brain barrier and act directly in the brain parenchyma, or alternatively act indirectly on the brain vasculature or by regulating the production of other factors in the periphery. Based on the study described above, VCAM-1 neutralization may be a beneficial strategy to reduce BEC inflammation and thereby preserve brain health^10^.

## Young bone marrow or HSC transplantation

It is possible that supplementation and/or reduction of multiple factors may be necessary to replicate the effects of heterochronic parabiosis, bone marrow transplantation and plasma transfer. If this is the case, a cellular therapy approach may be more effective. Analogous to the BMT approach we used in our mouse study^8^, this could involve bone marrow or HSC transplantation.

The main challenge in the clinical application of bone marrow or HSC transplantation would be the availability of young, suitably matched donors. Cord blood is a potential source of young HSCs, but supplies are limited, and two cords are currently required for transplantation in adults, although methods to expand cord blood stem cells are improving^17^. An alternative strategy may be to rejuvenate a patient’s own cells by reprogramming them to pluripotency (induced pluripotent stem cells, iPSCs), which would erase aging-associated epigenetic marks, and then re-differentiate iPSCs to HSCs. This approach would provide autologous cells to avoid the challenges associated with finding a matched donor or using mismatched donor cells. Methods to efficiently generate fully engraftable human HSCs from pluripotent stem cells have been demonstrated but still require optimization^18–20^. Another key question is how long the effects of transplantation would persist. It is currently unclear how quickly the transplanted young HSCs would age in the recipient’s aged bone marrow niche.

## Delivery of specific subsets of differentiated hematopoietic cells

We anticipate that HSC engraftment and continued production of young hematopoietic cells is important. However, we used total bone marrow cells in our BMT study^8^, so it is also possible that differentiated cells present in the donor bone marrow sample may be responsible for the effects we observed. Monocytes and the macrophages they produce are potential candidates given their key roles in tissue repair and remodeling. Indeed, monocyte adoptive transfer promoted amyloid-β clearance and improved cognition in a mouse model of Alzheimer’s disease with limited engraftment of donor-derived cells in the brain^21^. We did not observe any donor-derived cells in the hippocampus, either 3 weeks or 6 months post-transplantation^8^, but it is possible that monocytes transiently entered the brain at earlier timepoints after transplantation. iPSCs could be used to derive monocytes or other relevant blood or immune cell populations.

## Rejuvenation of endogenous HSCs

Another attractive strategy would be rejuvenation of endogenous HSCs. Aging of hematopoietic cells is thought to result from both intrinsic changes and extrinsic effects of the aging microenvironment, and recent studies have demonstrated that it is possible to rejuvenate HSCs by targeting their niche^22–24^. For instance, reduced β3-adrenergic receptor signaling in mesenchymal stromal cells (MSCs) in the bone marrow niche underlies aging-associated changes in HSCs, and administration of a β3-adrenergic receptor agonist rejuvenated the aging niche and restored HSC function^22,23^. In ongoing studies, we are therefore investigating whether β3-adrenergic receptor agonist treatment also impacts cognitive function. Adoptive transfer of young or rejuvenated (iPSC-derived) MSCs might be an alternative approach to achieve niche rejuvenation.

## Concluding remarks

Young blood clearly has rejuvenating effects in aging mice. The challenge is how to translate this into a clinical product for humans. Exciting developments in procuring young blood products using iPSC technology, or in designing molecular interventions to neutralize pro-aging mechanisms or rejuvenate the hematopoietic system, may lead to novel clinical approaches. As our aging population increases and aging-associated diseases such as Alzheimer’s and Parkinson’s become more prevalent, these anti-aging approaches may have a major impact on these disorders.
